# A high‐throughput method of analyzing multiple plant defensive compounds in minimized sample mass

**DOI:** 10.1002/aps3.1210

**Published:** 2019-01-08

**Authors:** Chandra N. Jack, Shawna L. Rowe, Stephanie S. Porter, Maren L. Friesen

**Affiliations:** ^1^ Department of Plant Pathology Washington State University Pullman Washington 99164 USA; ^2^ Department of Plant Biology Michigan State University East Lansing Michigan 48824 USA; ^3^ School of Biological Sciences Washington State University Vancouver Washington 98686 USA; ^4^ Department of Crop and Soil Sciences Washington State University Pullman Washington 99164 USA

**Keywords:** microplate, peroxidase, plant defense response, polyphenol oxidase, protease inhibitors, protein quantification

## Abstract

**Premise of the Study:**

Current methods for quantifying herbivore‐induced alterations in plant biochemistry are often unusable by researchers due to practical constraints. We present a cost‐effective, high‐throughput protocol to quantify multiple biochemical responses from small plant tissue samples using spectrophotometric techniques.

**Methods and Results:**

Using *Solanum lycopersicum* and *Medicago polymorpha* leaves pre‐ and post‐herbivory, we demonstrate that our protocol quantifies common plant defense responses: peroxidase production, polyphenol oxidase production, reactive oxygen species production, total protein production, and trypsin‐like protease inhibition activity.

**Conclusions:**

Current protocols can require 500 mg of tissue, but our assays detect activity in less than 10 mg. Our protocol takes two people 6 h to run any of the assays on 300 samples in triplicate, or all of the assays on 20 samples. Our protocol enables researchers to plan complex experiments that compare local versus systemic plant responses, quantify environmental and genetic variation, and measure population‐level variation.

The ability to quantify plant molecular responses to herbivory over time and compare variation within populations is useful in many research areas from ecology and evolutionary biology to applied agricultural research. Unfortunately, current methods are expensive, time consuming, and typically require large tissue masses. Like many attempts to assay molecular responses, research is limited by the rigorous nature of quantifying subtle physiological changes. Previously developed assays are thus effective but unusable by many researchers due to financial constraints (i.e., lack of access to spectrophotometers or large quantities of reagents) or the need to conduct a given assay on a large set of samples. These constraints have been recognized and addressed in some instances, such as protein quantification (Olson and Markwell, 2007). With the development and widespread use of microplate readers, some assays such as protease inhibition and protein quantification have been scaled and optimized for smaller reaction volumes and larger sample numbers, resulting in better replication (Pande and Murthy, 1994; Olson and Markwell, 2007).

One of the earliest biochemical responses to herbivory is the production of reactive oxygen species (ROS) such as superoxide (O_2_^−^), hydrogen peroxide (H_2_O_2_), and hydroxyl radicals (HO^−^) after depolarization of the plasma membrane due to leaf damage (Maffei et al., 2012; War et al., 2012; Zebelo and Maffei, 2015). Both chemical treatments and mechanical wounding can elicit ROS production (Maffei et al., 2007). H_2_O_2_ production is used both as a local signal to induce the hypersensitive response when plants are subjected to mechanical damage and as a systemic signal for the induction of additional defense responses (Orozco‐Cardenas and Ryan, 1999). The presence and activity of ROS also results in the production of a group of enzymes, peroxidases (POD), that are upregulated to perform a diverse set of physiological processes such as metabolism of ROS, restructuring of cellular walls, cross‐linking of complex polymers, and other critical functions (War et al., 2012). Increases in POD activity also decrease the nutritional quality of leaf tissue, which significantly reduces the growth and development of insect larvae (Mithöfer and Boland, 2012). Finally, the presence of plant PODs in insect guts may also be toxic to insects (War et al., 2012).

Two additional compounds that are produced in response to herbivory are polyphenol oxidase (PPO) and protease inhibitors (Mithöfer and Boland, 2012; War et al., 2012). PPO is upregulated directly by the presence of herbivore‐associated signaling compounds such as methyl jasmonate (Koussevitzky et al., 2004). PPO breaks down diphenolic compounds to produce more reactive phenolic compounds that have anti‐insect activity once consumed (War et al., 2012). Protease inhibitors are small molecules that prevent proteolytic activity. In response to herbivores, plants will produce protease inhibitors to inhibit protein catabolism in insect guts, which can halt the degradation of proteins that may serve as precursors used for various physiological processes (Mithöfer and Boland, 2012).

Assaying multiple responses on individual samples is critical for understanding host responses because many defense responses are interrelated. A prime example of this is the direct relationship between ROS production and the induction of PODs. PODs such as glutathione peroxidase reduce H_2_O_2_, are induced in response to high levels of H_2_O_2_, and catalyze the oxidation of other molecules (Quan et al., 2008); measuring both peroxide and PPO in the same sample thus gives additional insight into this cellular process.

Reducing the total amount of tissue required for an expanded array of assays enables researchers to perform both small molecule and enzymatic assays during a given investigation by allowing two separate extraction buffers to be used, resulting in smaller amounts of tissues being assayed in more ways. This allows complex responses across large numbers of individuals to be analyzed within a single experiment. Early methods of protein quantification such as the Bradford method and the Lowry method were dependent on the use of a spectrophotometer and thus large sample volumes (Bradford, 1976). Currently, assaying for the production of small molecules requires severe buffering conditions that both inhibit and degrade proteins present in suspended tissue samples due to the presence of compounds such as trichloroacetic acid, which causes protein precipitation (Rajalingam et al., 2009), thus preventing the use of a single buffer. In addition, as with protein‐based assays, the large sample masses required as a result of large reaction volumes for current spectrophotometric techniques limit the total number of technical replications and assays that can be performed on a given sample.

Since the onset of the next‐generation sequencing revolution, many studies now use genomic data as evidence of variation. Although this has proven to be a highly useful tool, it is important to assess functional variation as well. Studies have indicated that transcript abundance does not necessarily match functional activity in tissue samples (Greenbaum et al., 2003). This finding and others like it (Shafer et al., 2015) highlight the limits of ‐omics‐based techniques and should be considered when assessing functional diversity in physical populations of organisms.

These problems ultimately result in researchers either (A) assaying single physiological changes induced by herbivory as a metric for general herbivory responses or (B) resorting to various ‐omics techniques that are often expensive and ill‐suited to provide detailed information regarding specific physiological responses. Paired together, assaying both small molecule production and enzyme production at a higher level of replication would allow for a more holistic assessment of herbivory‐associated plant immunity responses.

Here, we present a cost‐effective method to assay multiple molecular responses in small sample masses (Appendix 1). The assays include total protein content, POD, PPO, H_2_O_2_, and trypsin‐like protease inhibitors. Many defense responses can be assayed individually but require diverse tissue extraction methods that are mutually exclusive. For our purposes, we selected induced responses that were both diverse and able to be assayed from a common sample extract. We tested our method on leaves taken from *Solanum lycopersicum* L. pre‐ and post‐herbivory to show that our assays can quantify differential plant responses. *Solanum lycopersicum* is often used to test biochemical defense responses, which we used to compare data we generated using our method and published protocols. After validating our assays using *S. lycopersicum,* we tested our protocol using *Medicago polymorpha* L., a leguminous plant whose biochemical responses to herbivory have not been quantified. With trifoliate leaves that may weigh less than 50 mg, *M. polymorpha* is representative of a “non‐model” plant. This protocol paves the way toward more comprehensively assaying plant biochemical responses to herbivory in non‐model plants and allows for greater sample capacity, which would allow for improved statistics, time course experiments, and more complex experimental designs.

## METHODS AND RESULTS

### Tissue preparation

To compare our protocol to current spectrophotometer protocols, we used tomato (*Solanum lycopersicum*, ecotype M82*)*, a model plant often used for testing defense responses, and the non‐model plant *Medicago polymorpha*. Tomato seeds were scarified with 600‐grit sandpaper, imbibed in dH_2_O for three days at 4°C in the dark to stratify, then placed in a dark cabinet overnight. Germinated seedlings were grown for three weeks in a grow room before inducing defensive responses. Burr medic (*M. polymorpha*) seeds (Appendix 2) were scarified as described above and planted into 158‐mL pots filled with Sungro Sunshine Mix #1 (SunGro Horticulture, Quincy, Michigan, USA). Plants were inoculated a week after planting with a rhizobium strain mixture of 10^7^ cells of equal parts *Ensifer medicae* strain WSM419 and *E*. *meliloti* strain 1021 to prevent nitrogen starvation and to mimic natural conditions.

To ensure that our protocol could adequately detect plant defense responses both pre‐ and post‐herbivory, we allowed soybean loopers (*Chrysodeixis includens*) to feed on leaves and also manually induced plant responses using caterpillar regurgitant to account for variable insect feeding patterns. The soybean looper regurgitant was generated by compressing stomachs with forceps post‐feeding on corresponding host plants. Leaves were manually wounded with scissors dipped in this regurgitant for both the *S. lycopersicum* and *M. polymorpha* assays. Leaf samples were taken from each plant at 0 and 24 h and flash frozen in liquid nitrogen for storage at −80°C until processing.

### Sample size and assay replication

All microplate and spectrophotometric assays were carried out using one leaf from five tomato plants (five biological replicates) of the same ecotype (M82). Spectrophotometer measurements were pooled, as is standard for those assays, for five technical replicates. An additional tomato plant, ecotype M82, was used to run the POD and PPO validation tests. Spectrophotometer validation tests were run in triplicate technical replicates. We used 10 *M. polymorpha* plants, each a different genotype (Appendix 2), to test our microplate protocol. None of the microplate samples (tomato, validation, *M. polymorpha*) were pooled, and each was run in triplicate for technical replicates.

### Assays

One challenge of attempting to assay multiple enzymes and small molecules from a single sample is finding an appropriate extraction buffer that will preserve the integrity of the metabolites while not creating conditions inhibitory for other assays. We were able to utilize two extraction buffers: a trichloroacetic acid (TCA) buffer and a protein extraction (PE) buffer. The TCA buffer provides the appropriate conditions for assaying the production of hydrogen peroxide (Junglee et al., 2014). The PE buffer was designed to provide the best crude extraction without the presence of interfering compounds. Phenylmethane sulfonyl fluoride (PMSF), the serine protease inhibitor commonly present in protein extraction buffers (Grimplet et al., 2009), was removed due to the need to assay the production of trypsin‐like protease inhibitors. β‐mercaptoethanol, also a common protein buffer ingredient (Grimplet et al., 2009) used as a reducing agent to ensure analysis of strictly monomeric proteins, was removed due to interference with the Thermo Scientific Pierce BCA Protein Assay Kit (Thermo Fisher Scientific, Waltham, Massachusetts, USA). Previous studies, specifically ones from which we modified original assays (Cavalcanti et al., 2004; Goud and Kachole, 2012), used extraction buffers lacking protease inhibitors and/or reducing agents with no significant change to final results. Our PE buffer thus results in a crude extract that provides predictable results when published assays were replicated for validation purposes (Table 1).

**Table 1:** Comparisfectrophotometer and microplate assays for peroxidase (POD) and polyphenol oxidase (PPO) of *Solanum lycopersicum* plants. By not pooling tissue samples, we are able to decrease replicate experimental error

Assay	Experimental mean^a^		Experimental SE^b^	
	Pre‐herbivory	Post‐herbivory	Pre‐herbivory	Post‐herbivory
POD spectrophotometer	1.17	46.8	68.14%	34.85%
POD microplate	6.31	256.47	3.34%	1.97%
PPO spectrophotometer	0.79	34.64	26.88%	26.74%
PPO microplate	1.98	122.26	14.6%	10.15%

a Values expressed as absorbance per gram fresh weight.

b Values expressed as percentage of mean.

Frozen leaf tissue from each plant was placed into two microcentrifuge tubes and weighed. The tubes were homogenized for 15 min at 30.0 Hz in a tissuelyser (QIAGEN TissueLyser II; QIAGEN, Germantown, Maryland, USA). The tube holders were made of Teflon and stored at −80°C. All samples and holders were also dipped in liquid N_2_ before homogenizing. One tube received 1 mL of the 0.1% TCA buffer, while the other received 1 mL of the PE buffer (1 mM EDTA, 88 mM Trizma base [Sigma‐Aldrich, St. Louis, Missouri, USA], 10% glycerol). Tubes were centrifuged at 4°C for 10 min at 17,000 × *g* in an accuSpin Micro 17 centrifuge (Thermo Fisher Scientific), and the supernatant was pipetted into clean tubes. The PE extract samples were then diluted to 1/10×. All absorbance values were run on a SpectraMax M2 combination spectrophotometer and microplate reader (Molecular Devices, San Jose, California, USA) and standardized for fresh weight. A detailed description of our protocols can be found in Appendix 1.

#### Peroxidase activity

POD activity was measured in triplicate for each sample and also included a tissue‐specific control. Wells of the microplate designated as treatment wells received 143 μL of POD reaction buffer (100 mM sodium phosphate buffer [pH 6.5] containing 5 mM guaiacol). Control wells received 143 μL of 100 mM sodium phosphate buffer (pH 6.5). A total of 25 μL of supernatant (enzyme source) was added to each well. We then added 32 μL of 5 mM H_2_O_2_ (final concentration 0.8 mM) to start the reaction. Plates were incubated in the dark for 15 min at room temperature before reading absorbance values at 470 nm.

#### Polyphenol oxidase activity

PPO activity was also measured in triplicate per sample (biological replicate) with a tissue‐specific control. Sample wells received 115 μL of 100 mM sodium phosphate buffer (pH 6.8) and 60 μL of 50 mM pyrocatechol. Control wells received 175 μL of 100 mM sodium phosphate buffer (pH 6.8). A total of 25 μL of supernatant (enzyme source) was added to all wells. Samples were incubated for 5 min before reading absorbance values at 420 nm.

#### Hydrogen peroxide (H_2_O_2_) quantification

The hydrogen peroxide quantification assay (H_2_O_2_) was implemented with few modifications. The primary change was to the measurement wavelength. As measured in the original protocol (Junglee et al., 2014), the triiodide produced as a result of the reaction mechanism has optimal absorbance at 285 nm with significant differences able to be determined at wavelengths up to 410 nm. Sample aliquots were taken from the 0.1% TCA buffer extraction. Sample wells received 100 μL of 1 M potassium iodide, 50 μL of 10 mM potassium phosphate buffer (pH 6.5), and 50 μL of sample aliquot. Control wells received 100 μL of dH_2_O, 50 μL of 10 mM potassium phosphate buffer (pH 6.5), and 50 μL of sample aliquot to account for tissue coloration. Samples were incubated in the dark for 20 min at room temperature. A standard curve was prepared by preparing wells with 100 μL of 1 M potassium iodide, 50 μL of 10 mM potassium phosphate buffer (pH 6.5), and 50 μL of 0.1% TCA, and then seeding with known amounts (5–20 nmol) of H_2_O_2_ (Appendix 3). Absorbance was measured at 390 nm and values were compared to the standard curve for quantification in nanomoles.

#### Compatible assays: Protease inhibition activity and protein quantification

To demonstrate the ease of implementing additional assays with our microplate methods described above, protein quantification and protease inhibition were performed on our samples as representatives of additional useful assays for surveying plant responses to herbivory samples and to test the efficacy of our protein extraction buffer.

Total protein content was measured using the Thermo Scientific Pierce BCA Protein Assay Kit (product number: 23337; Thermo Fisher Scientific) according to manufacturer instructions for microplate, but our general extraction buffer ensures that other protein quantification methods (e.g., Bradford, 1976; Peterson, 1977) can also be used.

Protease inhibition activity was quantified using an adapted method from Orians et al. (2000), in which activity is represented by the inhibition of trypsin. This assay requires the preparation of two reaction buffers per sample. Reaction buffer 1 was prepared in tubes with 133.3 μL of Trizma base buffer (Sigma‐Aldrich), 83.3 μL of 2% azocasein dissolved in Trizma base buffer, and 33.3 μL of 0.001 M HCl solution containing 200 ng of trypsin. Reaction buffer 2 was the same as reaction buffer 1, but additional Trizma base was substituted for the trypsin solution. A total of 100 μL of the sample extract was added to each tube. These serve as the sample measurement tube and the sample control tube. Reaction buffers 1 and 2 were used for positive and negative assay controls, respectively. The assay controls received 100 μL of Trizma base instead of enzyme source. All tubes were incubated at 30°C for 25 min. Post‐incubation, 133 μL of 100% w/v TCA was added, and tubes were centrifuged at 6146 × *g* for 10 min. After centrifugation, 100 μL of the supernatant were added to wells of a microplate that contained 100 μL of 1 M NaOH and absorbance was measured at 450 nm. As with the other assays, samples were run in triplicate.

### Protocol validation

The success of our protocol hinges on three points that we address through different validation methods. First, we validated that our assay is able to accurately quantify the same amount of enzyme activity compared to assays run using a spectrophotometer. We mainly focused on POD and PPO, the two enzymes assayed given these were the most modified protocols. Implementation of published protocols (Orians et al., 2000; War et al., 2011) on *S. lycopersicum* provided us with a point of reference for comparison of our modified methods. By first establishing an expected response to a given treatment, we are able to determine if the measured microplate response is sufficiently similar and reproducible. Both assays underwent similar modifications during the scaling process. Previous protocols required between 0.025 mL and 0.100 mL of 1× crude extract to be assayed in a final volume of between 2.5 mL and 3.1 mL of solution (Cavalcanti et al., 2004; Goud and Kachole, 2012). When scaling our total assay volumes down to fit the requirements of a standard 96‐well microplate, the volumes were reduced ~100‐fold.

**Figure 1:**
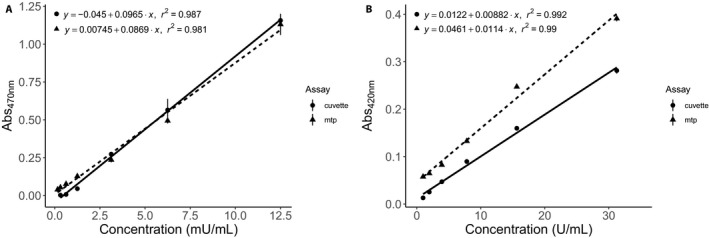
Comparison of absorbance values for peroxidase (A) and polyphenol oxidase (B) when measured using either a spectrophotometer (cuvette) or microplate reader (mtp) to generate a standard curve using horseradish peroxidase for peroxidase (measured at 470 nm) and mushroom polyphenol oxidase for polyphenol oxidase (measured at 420 nm). Line equations and *r* ^2^ values were generated by fitting data using a linear model. Each data point represents mean ± standard error. All concentrations were done in triplicate.

For each assay, we generated standard curves from enzymes obtained from Worthington Biochemical Corporation (Lakewood, New Jersey, USA). Horseradish POD with an activity of 220 U/mg dry mass was diluted to a stock concentration of 100 mU/mL in PE buffer. Standard curves were used to verify that the protocol was detecting analyte quantities within the detection limits of the machines used for absorbance measurements (Fig. 1). Serial dilutions were performed to obtain the following concentration values: 100 mU/mL, 50 mU/mL, 25 mU/mL, 10 mU/mL, 5 mU/mL, 2.5 mU/mL, 1.25 mU/mL, 0.625 mU/mL, and 0 mU/mL.

Mushroom PPO with an activity of 630 U/mg dry mass was diluted to a stock concentration of 100 U/mL in PE buffer. Serial dilutions were performed to obtain the following concentration values: 1000 U/mL, 500 U/mL, 250 U/mL, 125 U/mL, 62.5 U/mL, 31.625 U/mL, 15.625 U/mL, 7.81 U/mL, 3.91 U/mL, 1.95 U/mL, 0.977 U/mL. Absorbance values were measured using the SpectraMax M2 (Molecular Devices), and the *r* ^2^ values are similar for both standard curves (Fig. 1). This suggests that both the microplate and spectrophotometer are able to accurately predict concentrations given an absorbance due to the high *r* ^2^ values.

**Figure 2:**
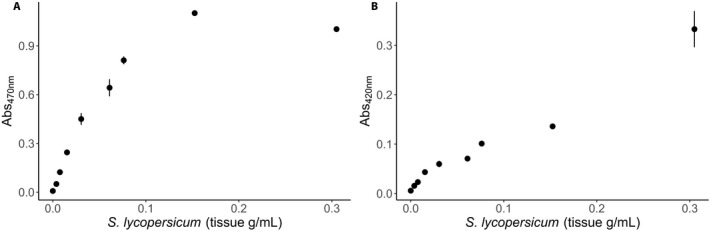
Serial dilutions of uninduced *Solanum lycopersicum* tissue. We serially diluted a homogenized tissue sample initially at a concentration of 0.38 grams fresh weight per milliliter to determine the lower limit of detection for the peroxidase (A) and polyphenol oxidase (B) assays. We used uninduced tissue with low expression of defense compounds and measured absorbance at 470 nm for peroxidase and 420 nm for polyphenol oxidase. Each dilution was measured in triplicate; data points shown are mean ± standard error.

Second, we confirmed that our tissue mass was sufficient for observing changes in the plants. Given the large quantities of tissue that are required for spectrophotometric‐based assays, researchers are often forced to pool tissue samples from different plants. Pooling tissue samples can increase variability because the sample pool contains multiple individual plant responses; this provides a strong argument for assaying individual plants (Zhang and Gant, 2005). Our microplate protocols require much smaller quantities of plant tissue, allowing us to measure each plant individually. We show that there is significant variation in expression both pre‐ and post‐herbivory between the five tomato plants of the same ecotype used in all five assays (Appendix 4, Table 2) even when they were grown in the same environment. This variation highlights the strength of our assay, which does not require plant tissue to be pooled. This is important because it indicates that our readings are more reproducible than alternative methods (Table 1). The difference in absorbance means between the microplate method and the spectrophotometric method is not of concern because differences can be explained as a consequence of using different detection methods. When we compared the absorbance values per plant sample between the microplate and spectrophotometer, the Pearson correlation coefficient was significant for both POD and PPO (POD: *r* = 0.931, *P* < 0.001; PPO: *r* = 0.920, *P* < 0.001). However, researchers are limited in what they can measure using spectrophotometers if their focal plant does not develop large or many leaves. We serially diluted tomato tissue to measure the lower limits of detection for our POD and PPO microplate assays and found that we were able to detect expression in as little as 3.8 mg of tissue. This was determined by doing a series of dilutions on a tissue sample to determine the linear range of the microplate assay (Fig. 2).

**Table 2:** ANCOVA table comparing absorbance values of *Solanum lycopersicum* tissue samples post‐herbivory using pre‐herbivory absorbance values as a covariate. Using the partial eta squared measure (*η*_*p*_^2^), we show that differences in absorbance values are mainly due to differences in individual plant responses, which shows that there is significant variation between plants of the same ecotype that were grown in the same environment

Assay	Grouping variable: Plant			Covariate: Pre‐herbivory value		
	*F*	*P*	*η*_*p*_^2^	*F*	*P*	*η*_*p*_^2^
Protein quantification	2897.86	<0.001	0.999	0.836	0.348	0.085
H_2_O_2_	12177.73	<0.001	0.999	0.0816	0.782	0.009
Peroxidase	120.46	<0.001	0.982	0.463	0.513	0.049
Polyphenol oxidase	14.71	<0.001	0.867	1.44	0.261	0.138
Protease inhibitor	14.5	0.012	0.935	3.981	0.117	0.499

Third, we confirmed that our wavelength used for H_2_O_2_ quantification was appropriate. We selected 390 nm for three reasons: (1) Previously published H_2_O_2_ assays (Velikova et al., 2000; Junglee et al., 2014) were able to quantify differences at wavelengths of 285 nm, 350 nm, and 390 nm. (2) We ran a spectral scan that indicated no significant differences in absorbance values at 390 nm relative to absorbance at 350 nm (*t*_(5)_ = −1.608, *P* = 0.169). (3) Both 285 nm and 350 nm are in the ultraviolet range, which means that assaying samples requires special plates to avoid issues of interference from the standard polymers used in 96‐well plates.

Finally, we ran all of our assays on trifoliate leaves (ranging in mass from 12 mg to 56 mg) collected from *M. polymorpha* as proof of concept that we could detect expression in actual small leaf tissue samples (Appendix 5).

## CONCLUSIONS

The measurement of plant biochemical variation in response to insect herbivory previously faced substantial limitations that have hindered the progress of the field. In particular, current practice in many labs is to use a single ecotype to measure differences between experimental treatments and to pool tissue from multiple leaves and individuals to obtain sufficient sample mass (War et al., 2011; Rajendran et al., 2014; Ferrieri et al., 2015). However, this approach has precluded the study of variation within and between individuals, which is what is relevant for real‐world interactions (Whitham, 1983; Winn, 1996; Bolnick et al., 2011). In contrast to previous techniques (Orians et al., 2000; War et al., 2011; Junglee et al., 2014), our protocol offers the ability to implement multiple assays on a large sample set by consolidating sample preparation buffers and running all assays on a microplate reader. This not only dramatically reduces the amount of tissue needed for a given assay, but also reduces the total time required to perform a given assay set. Between two researchers, we were able to perform each microplate assay on 300 samples in triplicate (900 reactions) in less than 6 h. In contrast, it took approximately 1.5 h to run the spectrophotometer protocols on 11 samples in triplicate (33 reactions). Thus, it would require 41 h to run the same 900 reactions using the spectrophotometer.

Using our high‐throughput protocols, researchers can now compare variation both within and between individuals, genotypes, and populations. Genetic and evolutionary biology studies often focus on variation between genotypes (e.g., Fitzpatrick et al., 2015; Kerwin et al., 2015), which requires the higher levels of experimental replication afforded by our method. Understanding the genetic variation associated with plant biochemical responses is critical both for understanding how coevolution has shaped these interactions, as well as for the success of molecular plant breeding for enhancing these interactions in agronomic settings. It is important to note that our protocol also enables paired measurements of multiple defense responses on the same tissue. This has several advantages over testing pools of tissue or defense responses on separate tissues—notably, we find high inter‐individual variation in biochemical responses within a single genotype of *S. lycopersicum*, underscoring how critical it is to perform paired assays. Furthermore, measuring the production of multiple metabolites and/or enzymes within a single sample will enable researchers to quantify tradeoffs in phytochemical production at the level of individual leaves, the scale at which insects interact with their plant hosts. Our protocol also enables researchers to compare systemic versus localized defense responses within the same plant, as multiple leaves can be assayed in parallel. Moreover, increased biological replication provides researchers the opportunity to test hypotheses with enhanced statistical power.

## AUTHOR CONTRIBUTIONS

C.N.J. and S.L.R. conceived of and designed the study in discussion with M.L.F. C.N.J. and S.L.R. performed the experiments. C.N.J. analyzed the data. S.S.P. contributed with sample preparation. C.N.J. and S.L.R. drafted the manuscript. C.N.J., S.L.R., S.S.P., and M.L.F. provided critical feedback and revisions to the manuscript. C.N.J., S.L.R., S.S.P., and M.L.F. gave final approval of the version to be published.

## APPENDIX 1 Protocol for analyzing multiple plant defensive compounds using a microplate reader.

**Buffers (all stored at room temperature)**

*Protein extraction (PE) buffer:*

4 mL of 25 mM EDTA (final concentration of 1 mM)

88 mL of 100 mM Trizma base (final concentration 88 mM; Sigma‐Aldrich, St. Louis, Missouri, USA)

8 mL of 80% glycerol (final concentration 10%)

*Trichloroacetic acid (TCA) extraction buffer:*

0.1% w/v TCA in H_2_O

**Reagents**

*Protein quantification assay:*

Pierce BCA Protein Assay Kit (Thermo Fisher Scientific, Waltham, Massachusetts, USA)

*Peroxidase (POD) assay:*

100 mM sodium phosphate buffer (pH 6.5)

5 mM guaiacol made in 100 mM sodium phosphate buffer (pH 6.5)

- May be liquid at room temperature; stock must be stored under inert gas (N_2_, Ar)
- Solution is light sensitive

5 mM H_2_O_2_

- 3% stock solution used; good for 4 weeks
- Solution is light sensitive

*Polyphenol oxidase (PPO) assay:*

100 mM sodium phosphate buffer (pH 6.8)

50 mM pyrocatechol

- Stock must be stored under inert gas (N_2_, Ar)
- Solution is light sensitive
- Solution only good for ~2 d
- Soluble in sodium phosphate buffer

*H*_*2*_ *O*_*2*_ *quantification assay:*

0.1% w/v TCA

1 M potassium iodide

10 mM potassium phosphate buffer (pH 6.5)

3% w/v H_2_O_2_ (0.988 M)

- Only good for 30 d
- Light sensitive and must be kept at 4°C

*Protease inhibition assay:*

100 mM Trizma base buffer (pH 7.8; Sigma‐Aldrich)

2% azocasein in Trizma base buffer (100 mM)

1 mM HCl solution (Trizma base) containing 200 ng of trypsin (0.1 mg/mL)

100% w/v TCA

1 M sodium hydroxide

**Extraction and homogenization**

1. Snap freeze harvested leaf tissue from each plant in microcentrifuge tubes and weigh.
2. Homogenize tubes for 15 min at 30.0 Hz in a tissuelyser (QIAGEN TissueLyser II; QIAGEN, Germantown, Maryland, USA) using Teflon‐coated adapters that are stored at −80°C to prevent additional accumulation of stress‐related compounds**.**
3. Add 1 mL of the 0.1% TCA buffer (Table A1) to microcentrifuge tubes with plant samples to be used for the hydrogen peroxide assay.
4. Add 1 mL of PE buffer (Table A1) to microcentrifuge tubes with plant samples to be used for all other assays.
5. Centrifuge tubes at 4°C for 10 min at 17,000 × *g* in an accuSpin Micro 17 centrifuge (Thermo Fisher Scientific) and pipette the supernatant into clean tubes. The PE buffer tubes are then diluted to 1/10×.

**Assays**

*Protein quantification*

Protein quantification was performed using the Thermo Scientific^ ^Pierce BCA Protein Assay Kit (product no. 23337; Thermo Fisher Scientific) according to manufacturer instructions for microplate samples. Because of the general nature of our buffer, other protein quantification methods (e.g., Bradford, 1976; Peterson, 1977) can also be used.

*Peroxidase (POD) activity*

1. Sample aliquots are taken from the 1/10× PE buffer extraction. All reactions are run in triplicate.
2. Create sample master mix by multiplying reaction components by the total number of reactions + 1. Reaction components are as follows: 143 μL of POD buffer (100 mM sodium phosphate buffer [pH 6.5] containing 5 mM guaiacol).
3. Create control master mix by multiplying reagent components by the total number of control reactions + 1. Reaction components are as follows: 143 μL of 100 mM sodium phosphate buffer (pH 6.5).
4. Aliquot 143 μL of each master mix (triplicate) to separate wells in a 96‐well plate.
5. Add 25 μL of supernatant (enzyme source) to each well and then add 32 μL of 5 mM H_2_O_2_ (final concentration 0.8 mM).
6. Incubate the plates in the dark for 15 min at room temperature.
7. Read absorbance at 470 nm on the microplate reader and express enzyme content as ([Abs_Spl_ – Abs_Ctrl_]/FW) (Abs/g). (FW denotes fresh weight.)

*Polyphenol oxidase (PPO) activity*

1. Sample aliquots are taken from the 1/10× PE buffer extraction. All reactions are run in triplicate.
2. Create sample master mix by multiplying reaction components by the total number of reactions + 1. Reaction components are as follows: 115 μL of 100 mM sodium phosphate buffer (pH 6.8) and 60 μL of 50 mM pyrocatechol.
3. Create control master mix by multiplying reagent components by the total number of control reactions + 1. Reaction components are as follows: 175 μL of 100 mM sodium phosphate buffer (pH 6.8).
4. Aliquot 175 μL of each master mix (triplicate) to separate wells in a 96‐well plate.
5. Add 25 μL of supernatant (enzyme source) to all wells and incubate for 5 min.
6. Read absorbance on the microplate reader at 420 nm and express enzyme content as ([Abs_Spl_ – Abs_Ctrl_]/FW) (Abs/g).

*Hydrogen peroxide (H*_*2*_ *O*_*2*_ *) quantification*

1. Generate a standard curve using a mix containing 100 μL of 1 M potassium iodide, 50 μL of 10 mM potassium phosphate buffer (pH 6.5), and 50 μL of 0.1% TCA per well. Spike each well with a known quantity of hydrogen peroxide from dilutions of 3% stock.
2. Sample aliquots are taken from the 0.1% TCA buffer extraction. All reactions are run in triplicate.
3. Create sample master mix by multiplying reaction components by the total number of reactions + 1. Reaction components are as follows: 100 μL of 1 M potassium iodide, 50 μL of 10 mM potassium phosphate buffer (pH 6.5), and 50 μL of enzyme source.
4. Create control master mix by multiplying reaction components by the total number of reactions + 1. Reaction components are as follows: 100 μL of dH_2_O, 50 μL of 10 mM potassium phosphate buffer (pH 6.5), and 50 μL of enzyme source.
5. Aliquot 200 μL of each master mix (triplicate) to separate wells in a 96‐well plate.
6. Incubate samples plus standard curve in the dark for 20 min at room temperature.
7. Read absorbance at 390 nm and compare values to the standard curve for quantification in nanomoles.

*Trypsin‐like protease inhibition activity*

1. Activity is represented by the inhibition of trypsin in sample aliquots taken from the 1/10× PE buffer. All reactions are run in triplicate.
2. Create sample master mix by multiplying reaction components by the total number of reactions + 1. Reaction components are as follows: 100 μL of enzyme source, 133.3 μL of Trizma base buffer (Sigma‐Aldrich), 83.3 μL of 2% azocasein dissolved in Trizma base buffer, and 33.3 μL of 0.001 M HCl solution containing 200 ng of trypsin.
3. Create sample control master mix by multiplying reaction components by the total number of reactions + 1. Reaction components are as follows: 100 μL of enzyme source, 166.6 μL of Trizma base buffer (Sigma‐Aldrich), and 83.3 μL of 2% azocasein dissolved in Trizma base buffer.
4. Create assay control master mix by multiplying reaction components by the total number of reactions + 1. Reaction components are as follows: 233.3 μL of Trizma base buffer (Sigma‐Aldrich), 83.3 μL of 2% azocasein dissolved in Trizma base buffer, and 33.3 μL of 0.001 M HCl solution containing 200 ng of trypsin.
5. Create a negative control by multiplying reaction components by the total number of reactions + 1. Reaction components are as follows: 266.6 μL of Trizma base buffer (Sigma‐Aldrich) and 83.3 μL of 2% azocasein dissolved in Trizma base buffer.
6. Incubate samples at 30°C for 25 min.
7. Post‐incubation, add 133 μL of 100% w/v TCA to all samples and centrifuge at 6146 × *g* for 10 min.
8. Aliquot 100 μL of 1 M NaOH to all wells of a 96‐well plate and then aliquot 100 μL of the supernatant to each well.
9. Read absorbance at 450 nm. Protease inhibition activity is calculated for pre‐ and post‐herbivory as 1 − ([sample absorbance − sample control absorbance]/[assay control absorbance − negative control absorbance]), standardized by tissue mass, and the values are reported as post‐herbivory minus pre‐herbivory.

**Table A1:** List of buffer conditions for each assay

Assay	Buffer	Dilution
Protein quantification	PE	0.1×
Polyphenol oxidase	PE	0.1×
Peroxidase	PE	0.1×
Protease inhibitor	PE	0.1×
Hydrogen peroxide	TCA	1×

## APPENDIX 2 *Medicago polymorpha* genotype with country and GPS coordinates.

W0419 (France; 43.618907, 4.813317), W0420 (Spain; 43.45713, 4.353194), W0077 (Spain; 43.301433, 2.344602), W0607 (USA; 43.221144, −123.406702), W0079 (France; 43.67624, 3.352244), W0076 (USA; 40.87011, −124.11282), W0517 (USA; 40.87011, −124.11282), W0603 (USA; 40.87011, −124.11282), W0146 (USA; 40.87011, −124.11282), W0421 (Turkey; 42.643558, 11.850325)
